# Encoding strategy affects false recall and recognition: Evidence from categorical study material

**DOI:** 10.2478/v10053-008-0130-0

**Published:** 2013-03-15

**Authors:** Justyna Olszewska, Joanna Ulatowska

**Affiliations:** 1 Department of Psychology, University of Michigan, USA; 2 Institute of Applied Psychology, Academy of Special Education, Warsaw, Poland

**Keywords:** false memory, imagery encoding, categorical study lists, mnemonic strategies

## Abstract

The present research investigated memory vulnerability to distortions. Different encoding strategies were used when categorized lists were studied. The authors assumed that an imagery strategy would be responsible for decreasing false memories more than a word-whispering strategy, which is consistent with the model of semantic access and previous research in the Deese-Roediger-McDermott paradigm (the DRM paradigm; Deese, 1959; Roediger & McDermott, 1995). A normative study of category lists and 4 experiments were conducted to verify the memory vulnerability to different encoding strategies (imagery, word-whispering, control). Half of subjects recalled and half recognized previously studied words. The results revealed a marked reduction in false recognition and recall after imagery encoding, relative to after word-whispering encoding.

## Introduction

People often think that we remember past events accurately; however, our memory is not always a faithful and exact representation of reality. Memory distortions, for example, schema-based memory errors (e.g., Bartlett, 1932; Holst & Pezdek, 1992), those that result from misleading post-event information (e.g., Davis & Loftus, 2007), and those that arise from associative memory structure (Deese, 1959; Dewhurst & Anderson, 1999; Roediger & McDermott, 1995), have been observed across different paradigms. In the present study, we focus on associative errors and test how different mnemonic strategies lead to different levels of memory accuracy.

The ease with which associative false memories may be induced is shown in one of the most heavily investigated procedures for studying memory confusions, the Deese-Roediger-McDermott paradigm (the DRM paradigm; Deese, 1959; Roediger & McDermott, 1995). Deese (1959) presented participants with lists of associated words and then asked them to recall the words. Apart from accurate recall, the participants also falsely recalled words that were strongly semantically related to those on the lists but were not presented (so-called *critical lures*). Roediger and McDermott (1995) replicated and extended Deese’s work and showed that the probability of erroneous recall of critical lures was similar to the probability of correct recall of words from the middle serial positions of the studied lists.

The effect of false memory caused by associative memory structure is also visible in the category repetition procedure which uses words that belong to the same category (Dewhurst, 2001; Dewhurst & Anderson, 1999). Dewhurst and Anderson (1999) showed participants one, four, or eight words from the same semantic category and revealed that the rate of falsely recognized critical lures increased with the number of words studied. Moreover, they compared the quality of memory distortions by asking participants to make *remember-know* judgments for each item they classified as *old*. *Remember-know* judgments reflect the subjective state of awareness that accompanies episodic memory retrieval. This procedure was introduced by Tulving (1985). Participants give a *remember* response if they can recollect details of the item’s study presentation, and a *know* response if the item feels familiar, but they cannot consciously recollect its earlier presentation. It appeared that false *remember* and *know* responses to the non-studied category items increased with the number of items from the same category that were presented at encoding (*remember* judgments were at a rate of .09 for eight category members). To some extent, these findings are consistent with those obtained by Roediger and McDermott (1995), who found that their participants had specific recollections of the critical lures. In their study, *remember* judgments had a probability between .38 and .58 depending on study condition. The discrepancy in *remember* rates toward critical lures, between Dewhurst and Anderson’s (1999) and Roediger and McDermott’s (1995) studies, stems from different materials (category lists vs. the DRM lists) and from different procedures they used. However, increase of *remember* responses with the number of items presented at encoding clearly shows that generation of words’ associates takes place at encoding.

Several researchers have tried to describe the nature of associative memory errors by using different manipulations during encoding. Gallo and Roediger (2002) as well as McDermott and Watson (2001) found that in the DRM paradigm, slower presentation of the list decreased the number of *old* responses to critical lures. This may give subjects time to process items more deeply, leading them to code more item-specific information. At retrieval, the distinction between the real experience and thoughts is more elaborated and this additional information results in a more accurate source memory (Johnson, Hashtroudi, & Lindsay, 1993).

A decrease in false alarms in the DRM paradigm was obtained also when more distinct perceptual information was presented at encoding (Arndt, 2010; Israel & Schacter, 1997) or simply when the modality of the presentation of the lists switched from auditory to visual (Gallo, McDermott, Percer, & Roediger, 2001; Smith & Hunt, 1998), suggesting that critical lures lack the perceptual features that are typical of the studied items.

The main explanation for this effect is based on the *distinctiveness heuristic*, which is defined as a metamemorial process that subjects may use at the time of retrieval to help decide whether a test item has been studied (Israel & Schacter, 1997). More distinctive information (or processing information in a distinctive manner) increases encoding of item-specific information (Howe, 2006).

A relatively modest number of studies showed also that a reduction in false recall and recognition could be obtained by creating mental images of the words presented during the study. This strategy was adopted from therapeutic methods of guided imagery and mnemonic techniques (Newstead & Newstead, 1998), and it is argued that it might increase the distinctiveness of encoded material. In one of these studies, Foley, Wozniak, and Gillum (2006) explicitly asked participants to generate images of presented objects (experimental condition), or to implicitly elicit images by describing a function of each object (control condition). The explicit instruction had a strong effect on false memory rates. Gunter, Bodner, and Azad (2007) also found that, in general, utilizing imagery reduced false alarms and led to greater accuracy. Foley, Hughes, Librot, and Paysnick (2009) revealed the effects of false memory reduction on images of individual items as well as integrated subsets.

Although most of the studies suggested the beneficial influence of imagery encoding on memory performance, Newstead and Newstead (1998) did not find statistically significant differences between the imagery encoding group and the control group. The authors argued that, although imagery is an effective mnemonic strategy, it works only when highly bizarre images are created or when it is used systematically as part of a more general strategy (e.g., a so-called *pegword technique* or *the method of loci*). Also, Burns, Jenkins, and Dean (2007, Experiment 2) did not show any decrease of false recall or increase of studied items recall after relational imagery encoding. However, when an item-specific imagery task was applied, recall of the studied items increased and recall of the critical lures decreased compared to control and relational imagery groups.

These findings are clearly at odds with one another. The discrepancy might result on the one hand from slight differences in instructing participants to create images (see Burns et al., 2007) or, on the other hand, from differences in the ability to generate images. Mental images are internally-generated, and their quality will differ between individuals (Richardson, 1977). Although it is still unclear how to elicit the most vivid images, no one disputes that the role of imagery in reducing associative memory errors is crucial.

Despite existing evidence that imagery affects memory, the research so far has not focused on methods that suppress mental images. Such a comparison would clearly show the advantage of the imagery strategy itself. Moreover, it may also constitute a new interpretative framework. Therefore, the main goal of the present studies was to verify how the imagery strategy affects memory accuracy as compared either to the condition in which creating images is limited or to the condition in which participants do not receive any particular instruction. For the purpose of these studies, a new, word-whispering technique was developed. This technique is based on reading words quickly or repeatedly, without saying them out loud, but only whispering them. We assume that in these cases, the central executive (Baddeley & Hitch, 1974) gives priority to the verbal task, and the ability to create images may be constrained. Although there is no explicit evidence that this technique suppresses images entirely, it possibly makes them much more difficult to generate. This hypothesis is in accordance with studies by Brooks (1967), who reported a conflict between reading messages and imagining the scenes described by those messages. He stated that reading interfered with visualization and described this phenomenon as a *selective interference*. This interference arises when the performance of two similar tasks requires engagement of the same modality (e.g., written presentation and visual activity; for a review, see De Beni & Moe, 2003; De Beni, Moe, & Cornoldi, 1997).

A number of studies have also investigated the effect of word production on memory (e.g., Conway & Gathercole, 1987; Gathercole & Conway, 1988; MacLeod, Gopie, Hourihan, Neary, & Ozubko, 2010) and found superior retention of words that were said aloud compared to those that were read silently. MacLeod et al. (2010), for example, differentiated between saying words aloud, mouthing, and silent reading and revealed the benefit of saying words aloud and mouthing over silent reading. However, the production effect obtained by saying words aloud or mouthing them is limited to within-subject designs (MacLeod et al., 2010). The authors argued that the advantage of word production was due to enhanced word distinctiveness in mixed-lists designs. In the present studies, a between-subjects design is utilized to reduce this beneficial effect of vocalization.

The findings of previous experiments led us to conduct four experiments that tested the influence of encoding strategy on memory performance. Although previous research has engaged the DRM procedure, we utilized the categorical study lists (Park, Shobe, & Kihlstrom, 2005). We decided to use this stimuli material because words are not only associated but also equally concrete as compared to DRM lists. Thus, we see an advantage in using this kind of stimuli when imagery instructions are tested because it is much more probable that after being asked to create an image participants will simply “mentally see” the referent of the word as compared to the DRM stimuli which are not equally concrete. In the previous studies that used the DRM list and imagery instructions, the role of imagery was less certain. It cannot be said that participants create real images of abstract words (like of *occupation, awake*, etc.). Thus it is not clear what other processes may be engaged following imagery instructions in the DRM paradigm. We also assume that engaging the phonological loop (Baddeley & Hitch, 1974) by whispering all the words from the studied lists may to some extent interfere with the visualization of these words and, thus, with imagery. Therefore, we hypothesize that imagery encoding will lead to an increase of memory accuracy and will outperform word-whispering encoding and encoding without engaging any specific strategy (control condition). The accuracy of these strategies will be tested both for recognition memory, that is an ability to correctly decide whether a person has encountered a stimulus previously in a particular context as well as for free recall task, in which participants are presented with a sequence of items and subsequently are asked to recall them in any order (Baddeley, Eysenck, & Anderson, 2009).

## Experiment 1

### Method^1^

#### Participants^2^

Forty-two undergraduate students participated in the study. Their ages were between 21 and 27 years (*M* = 21.74, *SD* = 1.29). There were 25 women and 17 men. They were tested during the regular classes and were not rewarded for the participation.

#### Materials

The first phase of the research was to prepare stimulus materials. As the participants were not native English speakers, the English-language word association norms (e.g., Russell & Jenkins, 1954) and their simple translation into Polish language were not appropriate because of possible cultural differences. The aim of this normative study was to elaborate lists which contained the most representative words in six given categories (fruits, vegetables, clothes, animals, kitchen equipment, and computer equipment). Moreover, the words should have comparable imaginability. Twenty-six undergraduates, who did not participate in further experiments, were asked to choose the prototype word for each category. Then, the words that occurred most often were chosen to create the category lists.

The stimulus materials for the studies consisted of words from six categories that were prepared in the normative study. Each word list consisted of 12 common and easy to visualize instances of the category, excluding the most common instance, which served as a critical lure. Each participant received six lists to study. The recognition test that was created was similar to that by Roediger and McDermott’s (1995) and it included 42 items: 12 studied and 30 non-studied words. Within the non-studied items there were three types of word: six critical lures (prototypes, e.g., *apple*), 12 foil words weakly related to the lists (two per list; e.g., *orchard*), and 12 foil words unrelated to any items in the six categories (e.g., *fan*). The weakly related words were randomly drawn from those that appeared below 13th position in the association frequency rankings in the normative study. Each block of tests started with a studied word. The last word was the critical lure. The rest of the items were randomly mixed.

#### Procedure

The subjects were randomly assigned and tested in small groups. They were seated so that they would not disturb one another during the encoding phase. The participants were told that the experiment was designed to test their memory and that they would be presented with several 12-word lists to remember.

Each subject received a booklet with the instructions followed by the categorical study lists. Half of the subjects received imagery instruction, the other half were given word-whispering instructions. In the imagery condition, the subjects were told to read the words carefully only once, to create a vivid image of the referent of each word, and to memorize it. In the word-whispering condition, the subjects were told to quickly read each word once and whisper it quietly and to memorize it. As the subjects were not given any time limit for that task and they studied the lists at their own pace, the average time necessary for completing the task was measured. The words appeared in the booklet in 12 points Times New Romans black font. After reading the lists, there was a retention interval lasting 2 min. The subjects were asked to solve mathematical tasks using their own sheets and pencils. After completing these, the subjects received sheets with a recognition test and they were asked to indicate by signing whether each item was old (it had been seen earlier on one of the lists) or new (it had not appeared on the study lists). All words appeared in the same font as during the encoding phase and were randomly intermixed. There was no time limit for this task.

### Results

*T*-tests were utilized to measure the difference between the imagery and word-whispering groups in their reactions to different types of items. Our primary interest was in comparing the rate of false alarms involving critical lures. The results are listed in Table 1. Critical lures were incorrectly judged as old much less often in the imagery than in the word-whispering encoding condition, *t*(40) = 3.49, *p* < .001, *d* = 1.1.

**Table 1.** Mean Proportion of False Alarms and Hits as a Function of Encoding Condition

	Response type			
Encoding condition	False alarms towards lure	False alarms towards related foils	False alarms towards nonrelated foils	Hits
Word-whispering	.41(.20)	.07 (.08)	.04 (.06)	.69 (.19)
Imagery	.17 (.23)	.02 (.04)	.007 (.02)	.81 (.11)

*Note*. Standard deviations in parentheses.

The participants in the imagery group were also more accurate in responding old to studied items than those in the word-whispering group, *t*(40) = -2.54, *p* < .05, *d* = 0.80.

The difference between these two groups in the false alarms rate towards related and unrelated foil items was also tested. Both rates in all conditions were close to zero, but the imagery group was significantly less prone to judge related foil words as studied than the word-whispering one, *t*(40) = 2.42, *p* < .005, *d* = 0.79. A similar pattern of results was also obtained for unrelated foil items, *t*(40) = 2.63, *p* < .005, *d* = 0.73.

In this experiment, two different mnemonic strategies were provided. In general, it is evident that the imagery strategy makes people less prone to memory distortions and consequently makes their recognition more accurate.

## Experiment 2

In Experiment 1, we tested (false) recognition. In Experiment 2, we tested whether imagery had a similar influence on (false) recall. Although Deese (1959) showed a reliable effect of false recall, most research showed that subjects were accurate in recall (e.g., Roediger & Payne, 1985).

### Method

#### Participants

Fifty undergraduate students participated in the study during regular classes. Their ages were between 21 and 27 years (*M* =21.5, *SD* =1.16). There were 31 women and 19 men. They were not rewarded for the participation.

#### Materials and procedure

This experiment used the same words from six categories as the previous experiment. The same two conditions - imagery and word-whispering - were provided. The instructions and the mathematical ability test were also the same as in the previous experiment.

In the second phase of the experiment, participants were asked to recall as many previously memorized words as possible and to write them down. The time for this task was 2 min. As the recall test was conducted after encoding the whole set of lists rather than after each list (see Roediger & McDermott, 1995), the participants were reminded of the names of the categories.

### Results

The rate of correctly recalled words was significantly higher in the imagery condition (*M* =.63, *SD* =.15) than in the word-whispering group, *M* =.34, *SD* =.11, *t*(48) = 7.46, *p* < .001, *d* = 2.2.

Errors in recall were analysed and then classified by two trained coders to identify those that were the most closely associated with the list. Single discrepancies in coding were discussed and one version of classification was settled. None of the participants recalled an unrelated word. The mean proportion of recalled critical lures was computed. In the word-whispering condition, the probability of an incorrect recall of a critical lure (*M* =.08, *SD* =.06) was significantly higher than in the imagery condition, *M* =.01, *SD* =.01, *t*(48) = 5.32, *p* < .001, *d* = 1.68. The results confirm the hypothesis that creating a vivid image of an item is a possible way to decrease false recall.

### Discussion

The results from Experiments 1 and 2 are consistent with those obtained in previous studies that focus on associative memory illusions (e.g., Roediger & McDermott, 1995); however, the rate of falsely recalled and recognized items was rather low. This was probably because we utilized category lists of words in this research, and the recall test, instead of being provided after studying each list, was provided after the whole set of lists.^3^ The significant difference between the word-whispering and imagery conditions, however, implies that memory sensitivity to distortions changes along with the way of encoding. Thus it suggests that the material was elaborated properly. As stated above, the subjects memorized the lists at their own pace. The average encoding time^4^ for the imagery group (approximately 4.5 min) was longer than for the word-whispering group (approximately 3 min). Thus, the time taken for encoding could be the reason for the different results in these groups. The research described above, as well as that by Foley et al. (2006, 2009) used various imagery encoding strategies; however, the time necessary to encode words was not controlled. Thus, it could have been unequal between conditions, which might have had an impact on memory. Our purpose then was to establish the average time necessary to encode words in both groups. Thus, the subsequent experiments would attempt to verify how different encoding strategies affect memory, discounting the influence of different encoding times.

Basing on the average time necessary to memorize the lists in Experiments 1 and 2, we determined that a presentation lasting 4 s for each word would be long enough, either to create a vivid image or to activate a purely verbal representation of each word. This was based on the average time necessary to memorize the lists in Experiments 1 and 2. It is also consistent with the procedure applied by McCabe and Smith (2002), who used 2 and 4 s for encoding each word in their experiment. Moreover, in the subsequent experiments we used a control group that was given no instruction on how to encode the lists.

## Experiment 3

### Method

#### Participants

Seventy-six undergraduates participated in the study and were tested during regular classes. Their ages were between 19 and 27 years (*M* =21.31, *SD* =1.35). There were 45 women and 31 men. Fifty-one were assigned to the two experimental groups (25 to word-whispering condition and 26 to imagery condition) and 25 to the control group. They were not rewarded for the participation.

#### Materials

The same stimulus materials used for Experiments 1 and 2 were utilized in this study; however, the words were presented on a screen and their duration was controlled (4 s for each word with a 1-s break between words). The recognition test included 42 items and was identical to that used for Experiment 1.

#### Procedure

The subjects were randomly divided into three groups: two experimental groups and one control group. They were then tested in small groups and seated so that they could not disturb each other. The subjects were told that the experiment was designed to test their memory and that they would be presented with several 12-word lists to remember. In the imagery condition, the participants were instructed to create a vivid image of the referent of each word and to memorize it. They were also informed that they had 4 s to create each image. Between each word a blank screen appeared for 1 s. In the word-whispering condition, the subjects were asked to repeat words quickly, whispering them quietly until they disappeared (also 4 s). The procedure in the control condition was the same except that the participants were not given any instructions concerning the method of encoding. The study phase lasted 6 min.

After having been presented with the lists, the subjects were asked to solve a mathematical problem. The time permitted for this task was 2 min. After completing this, the participants received a recognition test. The test consisted of 42 items, some of which had already been presented during the study phase and some not. Among the non-presented items there were critical lures, items weakly associated with the words from the lists (related foils), and items that were neither present nor associated (unrelated foils).

### Results

Four one-way ANOVAs with the single between-participants variable encoding condition (with levels word-whispering, imagery, and control) and Duncan post hoc analyses were used to test the effect of the encoding strategy on (a) the false alarms rate for critical lures, (b) the false alarms rate for related foil items, (c) the false alarms rate for unrelated items, and (d) the correct recognition rate. First, the false alarms rate for critical lures was compared. As in Experiment 1, the false recognition of critical lures was found to be substantially lower in the imagery group than in the word-whispering group or the control group, *F*(2, 73) = 3.73, *p* < .05, η^2^ = .09. Moreover, correct recognition was lower in the word-whispering condition than in the control condition, *F*(2, 73) = 3.04, *p* = .05, η^2^ = .08. The results are displayed in Table 2.

**Table 2.** Mean Proportion of False Alarms and Hits as a Function of Encoding Condition

	Response type			
Encoding condition	False alarms towards lure	False alarms towards related foils	False alarms towards nonrelated foils	Hits
Word-whispering	.23(.19)^a^	.01 (.03)^a^	.003 (.01)^a^	.76 (.15)^a^
Imagery	.11 (.15)^b^	.009 (.02)^a^	.003 (.01)^a^	.83 (.19)^ab^
Control	.23 (.19)^a^	.02 (.07)^a^	.006 (.03)^a^	.88 (.13)^b^

*Note*. Standard deviations in parentheses. Means with different letter indices (in columns) differ significantly (Duncan test post hoc analysis).

Neither the false alarms rate for related foil items, *F*(2, 73) = 0.75, *ns*, nor that for unrelated foil items, *F*(2, 73) = 0.18, ns, differed across the condition and they were close to zero.

The results obtained by the imagery and word-whispering groups are consistent with those from Experiment 1 and confirm our hypo-thesis that imagining while encoding plays a crucial role in protecting against false memory. However, an unexpected result was obtained in the control group. The high rate of correctly recognized items (comparable to the imagery group), along with the high rate of erroneous old reactions to critical lures (comparable to the word whispering group), suggests that the subjects might have used specific encoding strategies that strengthened their memory trace for the studied items. However, these strategies were not sufficient to faultlessly differentiate between old and new items.

## Experiment 4

### Method

#### Participants

Seventy-four undergraduates participated in the study. Their ages were between 21 and 27 years (*M* =21.7, *SD* =1.47). There were 50 women and 24 men. Fifty-two subjects were randomly assigned to the experimental groups (28 to word-whispering condition and 24 to imagery condition) and 22 subjects to the control group. The subjects were tested during regular classes and were not rewarded for the participation.

#### Materials and procedure

As in Experiment 3, in the experimental groups two conditions - imagery and word-whispering - were utilized between-subjects, and in the control group the participants were not given any particular encoding instruction. The instructions, duration of presentation, and mathematical ability test were the same as in the previous experiment.

In the second phase of the experiment the subjects were asked to recall as many previously memorized words as possible and to write them down. They were again reminded of the names of the categories.

### Results

Two one-way ANOVAs with the single between-participants variable encoding condition (with levels word-whispering, imagery) were conducted between-subjects to test the differences between the study conditions in correct and false recall. The rates of correctly recalled words in the imagery, word-whispering, and control groups were not statistically different, *F*(2, 71) = 0.75, *ns*, and amounted to .44 (*SD* =.1), .47 (*SD* =.13), and .44 (*SD* =.12), respectively.

As in Experiment 2, all the erroneously recalled words were analysed and then classified by two trained coders to identify those that were the most associated with the list. None of the falsely recalled words was unrelated to any of the lists. In the word-whispering condition, the mean proportion of recalled critical lures (*M* = .03, *SD* =.03)was significantly higher than in the imagery condition (*M* =.006, *SD* = .01) and in the control condition, *M* =.01, *SD* =.01, *F*(2, 71) = 7.35, *p* < .001, η^2^ = .17. The difference between the imagery group and the control group was not significant.

These results partially confirm the hypothesis that vivid mental images may be responsible for fewer errors, which is consistent with previous research. The fact that there was no significant difference between the imagery group and the control group suggests that the spontaneous encoding of concrete words might to some extent evoke their images (Paivio, 1971). However, other specific processes may also be involved in items processing.

## General discussion

In the present studies, we investigated how imagery strategy affected memory performance and demonstrated its superiority over other methods, in which creating images was limited or not explicitly requested. Four experiments differing in retrieval condition (recall and recognition) and encoding duration (self-paced or fixed duration) were conducted to test these assumptions. The research contributes to the previous findings on reducing associative memory distortions by means of imagery techniques by adding data from categorical lists.

The results obtained consequently suggest that the imagery encoding strategy leads to more accurate memory performance than the word-whispering strategy. This supports previous findings on imagery encoding (e.g., Foley et al., 2006, 2009; Gunter et al., 2007), which assume imagery as a crucial factor in discrimination between studied and non-studied but strongly associated items. Moreover, imagery encoding may be treated as an active task leading to better memory (see Meijer & Van der Lubbe, 2011). It would mean that this kind of encoding engaged more active involvement because besides phonological and visual sensory codes participants also generated a pictorial code. However, the hypothesis that quick or repeated whispering of encoded words would suppress the items’ visualization did not find full support.

Experiments 1 and 3 revealed that in terms of hits, the imagery instruction led participants to be, in general, more accurate than the word-whispering group. This difference was, however, statistically significant only in Experiment 1. In Experiment 3, the proportion of hits did not significantly discriminate the participants applying imagery instructions from those in the word-whispering and control groups. This discrepancy between the experiments may stem from the different encoding duration set up in Experiments 1 and 3. In Experiment 1,subjects in both conditions were instructed to read words quickly and only once, thus the word-whispering group needed less time to encode the items than the participants in the imagery condition, where after reading each word they were supposed to create a mental image of its referent. Thus, the overall time necessary to encode lists in the word-whispering group of Experiment 1 was shorter than in the imagery group, as well as in Experiments 3 and 4. Therefore, in Experiment 1 not only the imagery strategy but also the longer encoding time could be responsible for accuracy. The influence of encoding duration is consistent with research by McDermott and Watson (2001) that showed that longer presentation duration led to a decrease of memory distortion.

Although there is no significant difference in hits between the control and the imagery group in Experiment 3, a tendency to be more accurate is visible in the control group. This result is surprising and requires a comment. The participants in the control group were not instructed to apply any specific way of encoding, therefore it is highly possible that 4 s for encoding each word was long enough to use personally preferred mnemonic strategy which they were familiar with and which was oriented towards the best memorizing of the studied items, but not towards detecting critical lures that are automatically retrieved due to spreading activation (Collins & Loftus, 1975). This is reflected in the high rate of false alarms the control group demonstrated. One possible explanation lies in Paivio’s (1971) dual coding theory, which states that mental images of encoded concrete items may be evoked spontaneously. Consequently, these items are processed separately in two different channels, creating separate representations for information processed in each of them, and next visual and verbal codes can be used during retrieval. In other words, the participants might have used their own memorizing strategies which for some of them might have been based on mental image creation and for others, on other processes. However, more research should be carried out to precisely identify these processes.

There is also no significant difference between the word-whispering group and the imagery group in terms of the rate of hits, although the mean rate in the former group is lower. Therefore, we can notice a gradation with the word-whispering strategy as the least accurate strategy in terms of hits, and the control group as the most accurate. As we stated above, the participants in the latter group might have used personally preferred strategies to improve their memory for studied items. This implies that the imagery strategy might not be the best for everyone in maximizing accuracy. This may be caused by individual differences in participants’ visualization abilities as well as by the reasons listed by Newstead and Newstead (1998), who pointed out that imagery might be efficient only when highly distinctive images are created or when used systematically as part of a more general strategy.

The proportion of correctly recalled items is relatively low (see Roediger & McDermott, 1995). This might be an effect of the procedure utilized in both of the present recall experiments which provided the test after studying all six lists rather than after each of the lists. The hypothesis that imagery encoding has a beneficial influence on memory accuracy was confirmed also for recall. The results of Experiment 2 revealed a higher rate of correct recall in the imagery group than in the word-whispering group. However, in Experiment 4 the pattern of correct recall changed, showing no differences between groups. Moreover, the rate of correctly recalled items in Experiment 4 was substantially lower in the imagery condition and higher in the word-whispering condition compared with the corresponding condition in Experiment 2. This discrepancy between Experiments 2 and 4 could again be a consequence of the different encoding duration in both studies, as mentioned above, and suggests that in both recognition and recall, the time taken to encode items is a crucial factor that, along with different encoding strategies, modifies the correctness of responses and the sensitivity to critical lures.^5^

Also in line with the hypothesis and previous studies (Foley et al., 2006, 2009; Gunter et al., 2007), in all experiments the lowest rate of false alarms was obtained in the imagery condition as compared to the word-whispering condition. It is argued that the participants used the distinctiveness heuristic at the time of the retrieval of information (Israel & Schacter, 1997). They encoded words visually and retrieved them in the same modality, thus they were probably able to use additional information from the image created at encoding. According to the source-monitoring theory (Johnson et al., 1993), if elements are similar and share common details this creates a difficulty in terms of remembering the appropriate source of the item. The visualized items are more detailed; therefore, during recognition, critical lures are ignored. This is because the critical lures do not have these perceptual elements and it is easier to reject them and treat them as items not presented in the lists. Because in all experiments we conducted the word-whispering group and the control group achieved a higher rate of memory errors comparing to the rate reached by the imagery group, this suggests that a vivid mental image is used at retrieval and helps in judging critical lures as new items.

Although the word-whispering strategy of encoding led, in both recognition experiments, to a substantially higher rate of false alarms than imagery encoding, in Experiment 3 the participants in the word-whispering group appeared to be susceptible to false alarms towards critical lures similarly to the control group. This does not seem to be consistent with the hypothesis stating that whispering of words suppresses the creation of mental images and decreases the false alarm rate compared to the control group. Although in recent research (Forrin, MacLeod, & Ozubko, 2012) the word-whispering strategy is considered to be one of the mnemonic strategies that improve memory for words relative to a silent word-reading strategy, little is said about its superiority in reducing associative memory errors. On the other hand, it is possible that whispering of words leads to spontaneous creation of images; however, they may not be as clear as when created following explicit imagery instruction. Thus, whispering of words does not preclude that critical lures may possess some perceptual features. However, better memory performance in the imagery condition may result from vivid encoding of studied items which is consequently helpful in monitoring the source of items in subsequent memory test. In other words, while encountering critical lures at test participants may be aware that they were not engaged in creating images for these items even if they experienced a slight image spontaneously. This problem should be addressed in future studies.

A slightly different pattern of results regarding false recall was revealed in Experiment 4. In accordance with the hypothesis, it was shown that the highest rate of falsely recalled items was obtained in the word-whispering condition as compared to both the imagery and control groups. The word-whispering condition appeared to be the most susceptible to semantic intrusions. This probably occurred because this technique, by engaging a phonological loop, on the one hand did not give the subjects a chance to apply specific memory strategies familiar to them and, on the other hand, suppressed the processing of additional items (e.g., creating images). This remains in accordance with Brooks’ (1967) selective interference theory. While memorizing, the subjects in the word-whispering condition were not able to process the items more extensively (e.g., by engaging visualization) because reading the words made them unable to convert words into any non-verbal form. Future studies should examine other techniques that might suppress spontaneous images. Such results might show more precisely the influence of mental images on semantic memory errors.

While previous studies have used the DRM paradigm to show associative memory distortions, the current experiments applied categorical study lists which contain concrete words that are equally easy to visualize. In contrast, the words in DRM lists are both concrete and abstract, which can to some extent make visualization more complicated and more difficult to monitor. Taken together, our findings showed evidence that imagery is a crucial factor in reducing associative memory errors and maximizing accuracy. However, the results of the control groups showed that individual mnemonic strategies may appear, in some conditions, comparably efficient. From a theoretical perspective, the data we report converge with other recent findings demonstrating the facilitative effects of imagery encoding on memory performance (e.g., Foley et al., 2006, 2009). Moreover, by introducing the word-whispering and control conditions we establish a new province in which to investigate associative memory errors and their relationship with different encoding techniques.

## Footnotes

1 The procedure of the studies was approved by ethical committee of Academy of Special Education in Warsaw, Poland.

2 The participants in all four studies were native Polish speakers and were undergraduate students in one of the universities in Warsaw, Poland.

3 We provided the recall test after the whole set of lists because our main objective was to test general recall and recognition, not to verify the impact of recall on a later recognition test (Roediger & McDermott, 1995)

4 It was determined basing on the duration of encoding of the first and the last participant finishing studying of the lists.

5 Subjects in Experiments 1 and 2 memorized items at their own pace. This means that each word presentation was not exactly equated but adjusted to individual needs.
